# New mechanism of kinetic exchange interaction induced by strong magnetic anisotropy

**DOI:** 10.1038/srep24743

**Published:** 2016-04-21

**Authors:** Naoya Iwahara, Liviu F. Chibotaru

**Affiliations:** 1 Theory of Nanomaterials Group, Katholieke Universiteit Leuven, Celestijnenlaan 200F, B-3001 Leuven, Belgium

## Abstract

It is well known that the kinetic exchange interaction between single-occupied magnetic orbitals (s-s) is always antiferromagnetic, while between single- and double-occupied orbitals (s-d) is always ferromagnetic and much weaker. Here we show that the exchange interaction between strongly anisotropic doublets of lanthanides, actinides and transition metal ions with unquenched orbital momentum contains a new s-d kinetic contribution equal in strength with the s-s one. In non-collinear magnetic systems, this s-d kinetic mechanism can cause an overall ferromagnetic exchange interaction which can become very strong for transition metal ions. These findings are fully confirmed by DFT based analysis of exchange interaction in several Ln^3+^ complexes.

Anderson’s kinetic exchange interaction^1^,^2^ is ubiquitous in magnetic molecules^3^,^4^ and insulating materials^5^,^6^. In particular, the kinetic mechanism has been found as dominant contribution to the exchange interaction in various transition metal compounds. The mechanism has been also often advocated as reason for orbital ordering in transition metal oxides with orbitally degenerate metal sites^7^, especially, in magnetoresistive manganese oxides^8^.

In all these cases the magnetic orbitals are real and the exchange interaction in the case of non-degenerate sites is described by Heisenberg Hamiltonian, 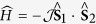. The kinetic exchange interaction originating from virtual electron transfer between single-occupied orbitals (s-s) is always antiferromagnetic^1^:


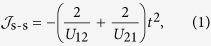


where *t* is the transfer parameter and *U*_*ij*_ is the electron promotion energy from site *i* to site *j*.

On the contrary, the electron delocalization between double-occupied and single-occupied orbitals (s-d) always results in a ferromagnetic contribution (the Goodenough’s mechanism^6^):


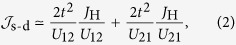


where *J*_H_ is the Hund’s rule coupling constant and *t* is the transfer integral between corresponding orbitals. Given the typical ratio 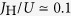
1,2, 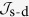 is by one order of magnitude smaller than 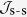. Then for comparable electron transfer parameters in s-s and s-d processes, the overall coupling is antiferromagnetic, 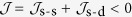.

A weak ferromagnetic interaction (2) is observed when the electron transfer between the single-occupied orbitals is negligible or zero, which is achieved for certain geometries of the exchange bridge^2^,^5^,^6^. Similar ferromagnetic contribution appears also for electron delocalization between single-occupied and empty orbitals, as well as in the case of degenerate magnetic orbitals (Kugel-Khomskii model)^7^. In all these cases the ferromagnetic kinetic contribution arises in the third order of perturbation theory after *t* and *J*_H_.

The kinetic exchange mechanism is equally important in *f* electron systems such as lanthanide and actinide compounds^9^,^10^,^11^,^12^. However its realization in these materials is expected to be different from transition metal compounds due to a more complex structure of multielectronic states on the metal sites, involving complex magnetic orbitals. The last are stabilized by strong spin-orbit coupling in lanthanides and actinides giving rise to unquenched orbital momentum in their low-lying multiplets^13^, which persists in any geometry of their environment. Unquenched orbital momentum also occurs in many transition metal complexes and fragments when the latter possess cubic^14^ or axial^15^ symmetry, and it was proved that its effects can persist also under significant deformations of the ligand environment^16^. Nonetheless, despite numerous examples of strongly anisotropic magnetic materials with unquenched orbital momentum on the metal sites, the basic features of kinetic exchange interactions in them have not been yet elucidated. In particular, despite the fact that the general form of the exchange Hamiltonian for strongly anisotropic system has been repeatedly derived in the past^11^,^17^,^18^,^19^,^20^, the nature of exchange parameter in Eq. (3) was not still discussed.

In this work the kinetic exchange interaction for metal sites with unquenched orbital momentum is investigated. On the example of strongly axial doublet states, we show that the paradigm of active magnetic orbitals as always belonging to half-filled ones does not hold for strongly anisotropic systems with unquenched orbital momentum on sites. In such systems the kinetic exchange interaction between single- and double-occupied orbitals is found to be of equal strength with conventional kinetic exchange interaction between single-occupied orbitals and can even make the entire interaction ferromagnetic. Contrary to the Goodenough’s mechanism (2), the s-d kinetic contribution found here appears already in the second order of perturbation theory being of the form (1).

## Results

### Doublets with unquenched orbital momentum

Metal ions are often characterized by non-zero orbital momentum 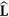
14,21. However, in order to keep (part of) it unquenched in complexes and crystals, the metal ions should also possess strong spin-orbit coupling which splits strongly the atomic (ionic) *LS* term in multiplets corresponding to definite total angular momentum 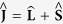
21. This is a standard situation in lanthanides and actinides^11^,^13^. Transition metal complexes in a threefold degenerate orbital state possess an unquenched orbital momentum corresponding to an effective 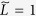
14. In this case the spin-orbit coupling leads to the formation of multiplets corresponding to total pseudo momentum 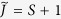, *S*, |*S* − 1|.

**Figure 1:**
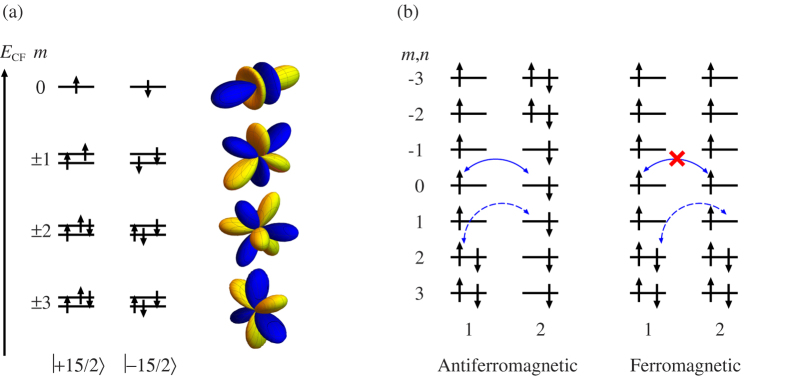
Electron transfer processes between doublets with unquenched orbital momentum. (**a**) Scheme of *f* orbital levels in axial crystal field. Numbers in the left side stand for the orbital angular momentum projection on the axis of the field. Pictures in the right side show the corresponding real orbitals (for *m* ≠ 0, only one of the two is shown). The electron configurations correspond to the wave functions of the Kramers doublet for Dy^3+^ with maximal projection of *J*. (**b**) Electron transfer processes between two collinear Dy^3+^ ions in axial Kramers doublets with maximal total momentum projection, *M*_*J*_ = ±15/2. s-s and s-d processes are shown by solid and dashed lines, respectively. *m*, *n* stand for orbital momentum projections on the direction of anisotropy axis on each metal site. Right plots correspond to reversed spin configuration on the site 2.

In low-symmetry crystal field, the (pseudo) *J*-multiplets on metal sites split into Kramers doublets in the case of odd number of electrons, or into singlets in the case of even number of electrons. The singlets in the latter case form quasi doublets for large *J* or perfectly degenerate (Ising) doublets in environments of axial symmetry^22^. In all these doublets the two wave functions are related by time inversion^21^. Besides, they are magnetic and contain a significant contribution of orbital momentum. Although no orbital momentum is conserved in these doublets, their *J*-multiplet genealogy implies large orbital contribution to the total magnetic moment. The latter necessarily implies that the magnetic orbitals and the wave functions of the doublets are complex. In the following we consider the simplest case of an axial crystal field, in which the atomic orbital wave functions preserve the projection of orbital momentum 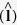 on the symmetry axis (*m*). The crystal-field orbitals are twofold degenerate with respect to the sign of the projection *m* and are described by the eigenfunctions |*l*, ±*m*〉 (Fig. 1(a)). For more than half-filled atomic orbital shell *l*^*N*^, *N* > 2*l* + 1, the ground atomic multiplet corresponds to *J* = *L* + *S* while the wave functions corresponding to the maximal projection, *M*_*J*_ = ±*J*, are represented by single Slater determinants. An example is the ground Kramers doublet of Dy^3+^ ion in strong axial crystal field shown in Fig. 1(a) 22. Further we consider this kind of axial magnetic doublets only, which allows us to describe the exchange mechanism in the simplest way, though the discussed effects are general for all doublets with unquenched orbital momentum. The doublets |*J*, ±*J*〉 correspond to a limit of strong axiality of the magnetic doublet states. For this axial doublets, the Zeeman interaction becomes strongly anisotropic with the main gyromagnetic factor *g*_*Z*_ ≠ 0 and *g*_*X*_, *g*_*Y*_ = 0^23^. Predominant axial components in the crystal field is a necessary condition to obtain axial doublet states. It is worth mentioning that the doublet states |*J*, ±*J*〉 appear quite often in the ground state of lanthanides and represent a great interest for the design of single-molecule magnets^23^.

### Exchange interaction for collinear doublets

The kinetic exchange interaction between doublet states is conveniently described by pseudospin formalism^21^, in which the doublet eigenfunctions |*J*, ±*J*〉 are put in correspondence to eigenfunctions |1/2, ±1/2〉 of an effective 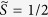. First, we consider the case of collinear doublets, when their main magnetic axes are parallel. Since one-electron transfer processes neither can switch nor mix the two doublet wave functions on each metal site, for relatively large *J*, the exchange Hamiltonian reduces to the following Ising form^24^:


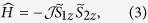


where 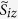 is the *z* component of the 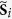, directed along the main magnetic axis on the corresponding metal site. In this case the exchange parameter 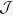 is simply derived from the difference between energies of antiferromagnetic and ferromagnetic configurations in Fig. 1(b), 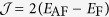. We calculated separately the contributions from s-s and s-d processes (Fig. 1(b)) to *E*_AF_ and *E*_F_ in the second order of perturbation theory after electron transfer. This yields the following contributions of s-s and s-d processes to the exchange coupling constant 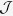:


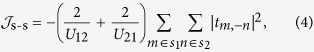



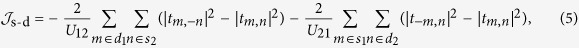


where *m* and *n* denote the orbitals on site 1 and 2, respectively, by corresponding angular momentum projections (Fig. 1(b)), and *s*_*i*_ and *d*_*i*_ indicate the sets of single- and double-occupied orbitals in the electron configuration |*J*, *J*〉 of site *i*, respectively. For example, for Dy^3+^ ion (site 1 in Fig. 1(b)) *s*_*i*_ = {−3, −2, −1, 0, 1} and *d*_*i*_ = {2, 3}. In these equations, *t*_*m*,*n*_ are electron transfer parameters between orbitals *m* and *n*. Note that we do not include effects ∝*J*_H_ (Goodenough’s mechanism) as being much weaker compared to the s-d contribution found here (*vide infra*).

While Eq. (4) looks as a standard expression for the s-s kinetic exchange parameter^1^,^2^, the s-d kinetic contribution, Eq. (5), does not appear for isotropic magnetic systems in this lowest order of the perturbation theory. We can see that it contains electron transfer terms of both signs, *i.e.*, antiferromagnetic and ferromagnetic contributions. The terms with *m* = 0 in the two brackets of Eq. (5) mutually cancel because of the relation |*t*_0,*n*_| = |*t*_0,−*n*_|. This is due to the real orbital corresponding to *m* = 0, for which we have 
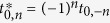
. Another evident cancellation occurs for terms with *n* = 0. The other pairs of terms in Eq. (5), with *m*, *n* ≠ 0, will not cancel each other unless the metal-ligand-metal fragment possesses special point symmetry. Therefore, for general geometry of exchange-coupled pairs, the s-d kinetic exchange is operative and represents a *new mechanism* of exchange interaction, proper to strongly anisotropic metal ions with unquenched orbital momentum only. The peculiarity of this mechanism is that it is of the order ~*t*^2^/*U*, *i.e.*, of similar strength as the s-s kinetic exchange, Eq. (4). However, at variance with the s-s kinetic exchange, the s-d exchange can be both antiferromagnetic and ferromagnetic as Eq. (5) shows.

Due to time-reversal symmetry the transfer parameters contributing to Eq. (5) satisfy the relations |*t*_*m*,*n*_| = |*t*_−*m*,−*n*_|. Using these relations, the total exchange parameter 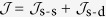 is obtained as


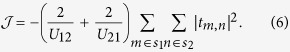


Despite its similar form to 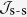 in Eq. (4), the above expression involves different orbitals in the second summation.

The kinetic exchange interaction between a strongly axial doublet and an isotropic spin is described by the same Ising Hamiltonian (3) in which one of the pseudospin operators is replaced by the real spin projection 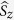 of the corresponding site. The expressions for the exchange parameters coincide with Eqs (4) and (5), in which the second summation runs over real orbitals (*a*) for isotropic spin site. Applying the same argument as for the orbital *m* = 0 in the previous case, we come to the relations |*t*_−*n*,*a*_| = |*t*_*n*,*a*_| which cancel the terms in each bracket of Eq. (5). Thus no s-d kinetic mechanism is expected in this case.

#### Assessment of 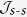 and 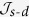 in lanthanide complexes

**Figure 2:**
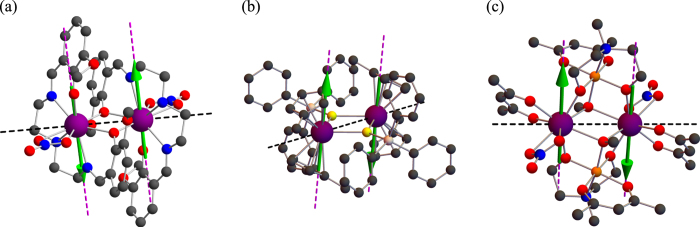
The structures of investigated binuclear lanthanide complexes Ln_2_. (**a**) Ln = Tb, Dy, Ho^25^, (**b**) Ln = Dy^26^ and (**c**) Ln = Dy^27^. Color legend: Ln purple, O red, C gray, N blue, S yellow, Cr orange and Si beige. The pink dashed line is the direction of the main magnetic axis and the green arrow is the magnetic moment on Ln ions calculated *ab initio*^25^,^26^,^27^.

To assess the importance of s-d contribution to the exchange interaction in real complexes, we performed a density functional theory (DFT) based analysis of 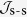 and 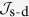 for several previously investigated lanthanide complexes^25^,^26^,^27^. To this end, we first made the localization of Kohn-Sham orbitals on the metal centers and the bridging ligand. This allowed us to extract the metal-ligand transfer parameters (the metal-metal ones turned out to be negligibly small in this approach). This tight-binding model together with the Hubbard repulsion energy (described by one single parameter *U* due to the equivalence of the metal sites, see Fig. 2) was downfolded on the ground spin-orbit doublet states of Ln ions (Fig. 1(a)). This allowed us to calculate straightforwardly the energies of ferromagnetic and antiferromagnetic configurations (Fig. 1(b)) and to obtain the corresponding total 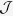 in Eq. (3). Then, repeating this procedure by blocking electron transfer processes from double occupied orbitals on the Ln sites, we obtain a net s-s contribution to the exchange coupling, 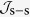, and finally the s-d contribution: 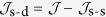. In this calculations, the parameter *U* was chosen to reproduce the experimental exchange parameter 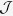 (Table 1).

The obtained s-s and s-d contributions are given in Table 1. The Dy and Ho complexes from isostructural series (a) and the Dy complex (b) show that the s-d contribution is by far not negligible in comparison with the s-s contribution. The increase of the s-d contribution with Ln atomic numbers in the isostructural series (a) is explained by the increase of the number of the double-occupied orbitals. On the other hand, the vanishing s-d contribution in the complex (c) is due to the cancellation of the ferromagnetic and antiferromagnetic contributions in the expression for 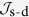, Eq. (5).

### Exchange interaction for non-collinear doublets

**Figure 3:**
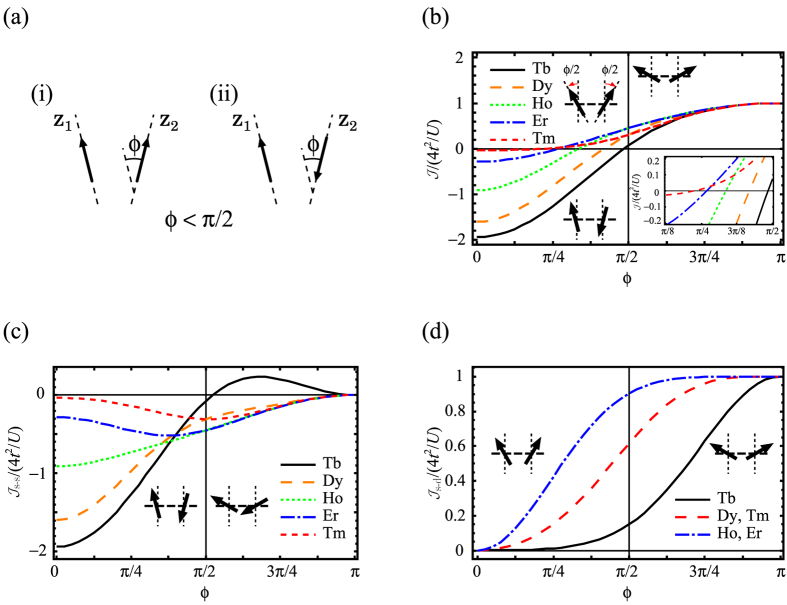
s-s and s-d exchange contributions in non-collinear system. (**a**) Definition of ferro (i) and antiferro (ii) ordering for non-collinear case. The main magnetic axes *z*_1_ and *z*_2_ are generally non-coplanar. (**b**) 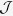, (**c**) 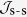 and (**d**) 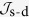 for Ln^3+^ dimers. in the symmetric exchange model (inset of plot (**a**)) as function of the angle *ϕ* between the local main magnetic axes. The only non-zero transfer parameter is 
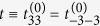
.

In non-collinear magnetic systems the main magnetic axes on sites make an angle *ϕ* (Fig. 3(a)). The new feature which appears in this case is that electron can transfer to an orbital of a neighbor site in both ferro and antiferro configurations (see the definition in Fig. 3), with the probability depending on *ϕ*. As in the collinear case, the single-electron transfer processes cannot switch the multiplet components, 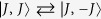, when *J* is sufficiently large^24^. Therefore, the exchange interaction will be described by the same Ising Hamiltonian (3) with the difference that now pseudospin operators describe momentum projections along corresponding main magnetic axes (*z*_1_ and *z*_2_ in Fig. 3(a)). The exchange parameter corresponding to s-s processes is obtained as


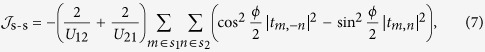


and contains now both ferro and antiferro contributions. On the other hand the exchange parameter for the s-d processes remains unchanged, Eq. (5). One can see from Eq. (7) that 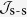 is not proportional to cos *ϕ* unless we have an additional condition |*t*_*m*,*n*_| = |*t*_*m*,−*n*_|. As was discussed above, the latter is fulfilled for interacting axial doublet and isotropic spin, in which case also the s-d contribution, Eq. (5), vanishes. One should note that the transfer parameters in Eqs (5) and (7) are defined for orbitals quantized along main magnetic axes on the corresponding metal sites and, therefore, are implicitly dependent on angle *ϕ*.

### Ferromagnetic kinetic exchange interaction

Contrary to collinear case for which 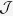 is always antiferromagnetic (Eq. (6)), the contributions 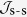 and 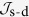 can be of either sign in non-collinear systems, so that the resulting exchange interaction can be both ferro and antiferromagnetic. To investigate this situation we consider a symmetric homonuclear dimer model. We assume that the electron transfer only takes place between one pair of orbitals, 
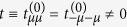
, where ±*μ* are orbital momentum projections on the common axis *z* connecting the metals. Figure 3(b) shows calculated 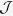 for *μ* = 3 as function of *ϕ*. For small angles, 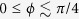, 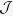 is always antiferromagnetic. In this domain 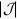 decreases with increasing *ϕ* and at some critical *ϕ*_*c*_ < *π*/2 becomes ferromagnetic. Remarkably, the magnitude of the ferromagnetic 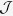 is of the order ~*t*^2^/*U* and its relative strength gradually increases when approaching the end of the lanthanide series. Figure 3(c,d) show the evolution of 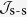 (7) and 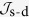 (5). For *ϕ* < *π*/2, the s-s and s-d processes stabilize the antiferro and ferromagnetic states, respectively. They are found in competition and the latter (s-d) begins to exceed the former (s-s) at a critical *ϕ*_*c*_, which has a simple explanation. The number of single-occupied orbitals decreases with the increase of the number of *f* electrons (*N*). Following this trend, the 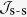 will always decrease with *N*. On the contrary, 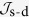 roughly depends on the multiplication of the number of single- and double-occupied orbitals. This is the reason why it first increases with *N* till *N* = 11 and then begins to decrease. As a result, the critical *ϕ*_*c*_ decreases with the increase of *N* and in the cases of Ho, Er and Tm complexes, the critical *ϕ*_*c*_ is as small as ca *π*/4. The reasons given above explain also the decrease of 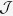 in the domain 0 < *ϕ* < *π*/2 when moving towards the end of the lanthanide series (Fig. 3(b)). For *ϕ* > *π*/2, the s-s and s-d processes tend to stabilize the ferro and antiferromagnetic states, respectively. In this domain the contribution from the s-d processes is dominant because the contribution from s-s processes gradually decreases with *ϕ* and becomes completely quenched at *ϕ* = *π*.

The change of the sign of kinetic exchange parameter is not specific only to lanthanides. Similar results are obtained for compounds with transition metal sites in axial ground doublet states with unquenched orbital momentum. We obtain again that in the domain *π*/4 < *ϕ* < *π*/2 the exchange parameter becomes ferromagnetic. Moreover, for *d*^7^ and *d*^8^ metal ions this can attain values of ~*t*^2^/*U*, which corresponds to a very strong ferromagnetic coupling for transition metal compounds (see Supplemental Materials).

#### Effect of non-collinearity on exchange coupling in Er_2_ complex

**Figure 4:**
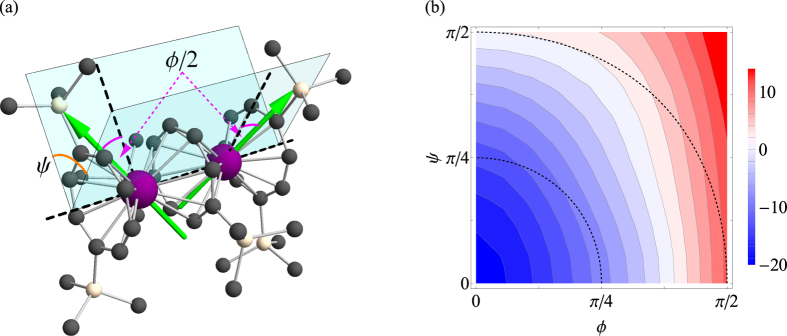
Ferromagnetic exchange interaction induced by strong magnetic anisotropy. (**a**) The definition of the angles *ϕ* and *ψ* defining the relative orientation in the Er_2_ complex with equivalent metal sites. (**b**) Variation of 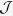 with respect to *ϕ* and *ψ*. The internal dashed line corresponds to the angle *π*/4 and the external to the angle *π*/2 between the magnetic axes. The blue and the red regions stand for the negative and positive values of 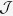, respectively (cm^−1^).

The evolution of the exchange parameter in function of the angle between the magnetic axes on metal sites is studied on the example of an Er_2_ complex^28^ (Fig. 4(a)). The calculation has been done in full analogy with the previous case of collinear Ln_2_ complexes (Fig. 2 and Table 1). Similarly to the model calculations (Fig. 3), with the increase of *ϕ* the antiferromagnetic exchange interaction becomes ferromagnetic around *ϕ*_*c*_ ≈ 2*π*/5 (Fig. 4(b)). The shift of *ϕ*_*c*_ in comparison with the model calculations is due to the existence of many electron transfer processes in this complex. In real systems, the direction of main magnetic axes could be controlled by varying ligand environment.

## Discussion

In this work, we investigated the kinetic exchange interaction between axial magnetic doublets with unquenched orbital momentum. We find a new mechanism of exchange interaction based on electron transfer between single- and double-occupied orbitals. Contrary to conventional spin systems, the s-d kinetic contribution found here is not related to Goodenough’s mechanism (2), arising due to the Hund’s rule coupling (*J*_H_) on metal sites, but due to the second-order kinetic mechanism (1). On this reason, this kinetic contribution is as strong as the conventional kinetic exchange between single-occupied orbitals but, at variance with the latter, can be ferromagnetic. In non-collinear magnetic systems the s-d kinetic mechanism can cause an overall ferromagnetic exchange interaction of the order of *t*^2^/*U*, starting from angles ~*π*/4 between main magnetic axes. These conclusions are fully supported by quantum chemistry based analysis of Ln_2_ complexes. The key feature underlying the new mechanism is that the double-occupied orbitals change under time inversion in strongly anisotropic sites due to unquenched orbital momentum. This is found in sharp contrast to the case of isotropic and weakly anisotropic sites, where no change of double-occupied orbitals occur under time inversion. The obtained results offer a new view on the exchange interaction in lanthanides, actinides and transition metal ions with unquenched orbital momentum. In particular, they show the way to achieve strong ferromagnetic coupling between metal ions, a long sought goal in magnetic materials^4^.

## Materials and Methods

The DFT calculations have been done with the ORCA package^29^, using B3LYP exchange-correlation functional^30^, in which the Hartree-Fock contribution to the exchange part was increased from 20% to 40%. This was done to reproduce the experimental isotropic exchange parameters in isostructural Gd_2_ analogues of investigated complexes. The derivation of tight-binding Hamiltonian for localized Kohn-Sham orbitals and the projection of the Hubbard model on the ground doublets of investigated Ln_2_ complexes is described in Supplemental Materials.

## Additional Information

**How to cite this article**: Iwahara, N. and Chibotaru, L. F. New mechanism of kinetic exchange interaction induced by strong magnetic anisotropy. *Sci. Rep.* **6**, 24743; doi: 10.1038/srep24743 (2016).

## Supplementary Material

Supplementary Information

## Figures and Tables

**Table 1:** The exchange coupling parameters 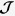, 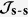 and 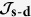 (cm^−1^) for strongly axial magnetic complexes (Fig. 2).

System	Ln	Ref.	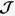	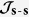	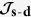
(a)	Tb	25	−3.57	−3.58	0.01
(a)	Dy	25	−2.97	−2.51	−0.46
(a)	Ho	25	−3.22	−1.84	−1.38
(b)	Dy	26	−2.20	−1.78	−0.42
(c)	Dy	27	−0.51	−0.51	0.00

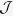
 corresponds to experimentally extracted exchange parameter. The exchange parameters 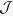 for a series of complexes (a) were obtained from experimental Ising parameter after extracting the magnetic dipole interaction.
