# Supplementary material for: New mechanism of kinetic exchange interaction induced by strong magnetic anisotropy
Source: Sci Rep. 2016 Apr 21;6:24743. doi: 10.1038/srep24743 (PMC4838874; doi:10.1038/srep24743)
Supplement: Supplementary Information [file srep24743-s1.pdf]

Supplemental Materials
for
New mechanism of kinetic exchange interaction induced by strong magnetic anisotropy

Naoya Iwahara and Liviu F. Chibotaru
Theory of Nanomaterials Group, Katholieke Universiteit Leuven, Celestijnenlaan 200F, B-3001 Leuven, Belgium
(Dated: January 28, 2016)

This material contains:
1) The lanthanide complexes and modeling for the electronic structures;
2) The density functional theory calculations;
3) The extraction of the Ising exchange parameters;
4) The s-s and s-d exchange mechanisms in the d metal complexes.

I. MODEL HAMILTONIAN FOR BINUCLEAR MAGNETIC SYSTEMS

As the anisotropic magnetic systems, we treat binuclear lanthanide complexes [S1–S4]. In order to describe the low-energy exchange states of these systems, the Hubbard Hamiltonian was used:

$$\hat{H} = \hat{H}_0 + \hat{H}_{\rm t} + \hat{H}_{\rm bi}, \tag{S1}$$

$$\hat{H}_0 = \sum_{r\sigma'} \epsilon_r \hat{n}_{r\sigma'}, \tag{S2}$$

$$\hat{H}_{\rm t} = \sum_{i=1,2}\sum_{m\sigma}\sum_{r\sigma'} \left(\tau_{mr}^{iL} D^{\frac{1}{2}}_{\sigma\sigma'}(R_i) \hat{c}_{im\sigma}^\dagger \hat{c}_{Lr\sigma'} + \tau_{rm}^{Li} D^{\frac{1}{2}*}_{\sigma\sigma'}(R_i) \hat{c}_{Lr\sigma'}^\dagger \hat{c}_{im\sigma}\right), \tag{S3}$$

$$\hat{H}_{\rm bi} = \sum_{i=1,2}\sum_{m\sigma\ne m'\sigma'} \frac{u_f}{2} \hat{n}_{im\sigma}\hat{n}_{im'\sigma'}. \tag{S4}$$

Here, $\hat{H}_0$, $\hat{H}_{\rm t}$, $\hat{H}_{\rm bi}$ indicate the orbital energy level of the bridging atoms with respect to the f orbital level, the electron transfer Hamiltonian between metal site and the bridging atoms (ligand), and $\hat{H}_{\rm bi}$ is the Coulomb repulsion on f metal sites, respectively. In the Hamiltonian, i $(= 1, 2)$ is the metal (lanthanide) center, L is the bridging ligand, m $(= 3, 2, ..., -3)$ is the projection of the orbital angular momentum of f atomic orbital, r is the orbital energy level of the ligand, σ, σ' is the spin projection, $\hat{c}_{im\sigma}$ ($\hat{c}_{im\sigma}^\dagger$) and $\hat{c}_{Lr\sigma}$ ($\hat{c}_{Lr\sigma}^\dagger$) are the annihilation (creation) operators on the $m\sigma$ orbital of metal site and $r\sigma$ orbital of the ligand L, respectively, $\hat{n}_{im\sigma} = \hat{c}_{im\sigma}^\dagger\hat{c}_{im\sigma}$ and $\hat{n}_{Lr\sigma} = \hat{c}_{Lr\sigma}^\dagger\hat{c}_{Lr\sigma}$ are the number operators, τ_{mr}^{iL} $(= \tau_{rm}^{Li*})$ is the transfer parameter between magnetic center and ligand, and u_f is the Coulomb repulsion energy on f metal site. Since the transfer parameter between metal sites are small, we neglect the direct electron transfer between the metal centers. The Coulomb repulsion on the ligand and the intersite one between the metal center and the ligand as well as the Hund's rule coupling are neglected because they are smaller than u_f. Based on the Hamiltonian, we describe the low-energy magnetic states. The transfer parameters are extracted from density functional theory (DFT) calculations and the Coulomb repulsions are determined to reproduce the experimental exchange parameters.

The model Hamiltonian Eq. (S1) is not the same as the model Hamiltonian used for the derivation of the exchange interactions in the main text. In the latter model Hamiltonian, the electron transfer between the metal sites and ligand is reduced to the transfer parameter between the metals. However, in the DFT based calculations of the exchange parameters, such simplification is not always possible because the f orbital levels and ligand levels are sometimes close to each other. Thus, we take Eq. (S3) for our calculations of the lanthanide complexes.

II. DFT CALCULATIONS

In order to find a density functional which is suitable for the electronic structure calculations of lanthanide complexes, we calculated (i) the exchange interactions and (ii) the binding energies with several methods (DFT and Hartree-Fock (HF) method), and compared the results with experimental data. We found that the hybrid functional including about 40 % of HF exchange is suitable to express one-orbital parameters (ϵ_r and τ_{mr}^{iL} in Eqs. (S2) and (S3)) of lanthanide complexes. For the quantum chemistry calculations, we used ORCA3.0 package [S5].

A. Isotropic exchange parameters for Gd^{3+} complexes

In general, the spin-orbit coupled ground states of the lanthanide ion (Dy^{3+}, Er^{3+}, etc.) cannot be adequately treated within single Slater determinant approach. On the other hand, the ground state of half-filled Gd^{3+} ion is in a good approximation described by pure spin state, and the state with maximal spin projection is a single Slater determinant. Thus, for the quantum chemistry calculations, we replaced the lanthanide ions (Dy^{3+} and Er^{3+} ions) by Gd^{3+} ions. As the functional, we chose B3LYP with various HF exchange contributions (20 -

60 %). The variation of the contribution of the HF exchange is to reduce the exaggerated electron transfer and to increase the underestimated Coulomb repulsion on site. The molecular structures were taken from the x-ray diffraction data. As the basis set, SVP was used.

The exchange parameter between metal sites was obtained within broken-symmetry approach [S6]. The exchange parameter $\mathcal{J}$ defined by Heisenberg Hamiltonian,

$$\hat{H} = -\mathcal{J}\hat{\mathbf{S}}_1 \cdot \hat{\mathbf{S}}_2, \tag{S5}$$

is estimated using the high-spin and broken-symmetry (low-spin) states:

$$\mathcal{J} = -2\frac{(E_{\text{HS}} - E_{\text{BS}})}{\langle\hat{\mathbf{S}}^2\rangle_{\text{HS}} - \langle\hat{\mathbf{S}}^2\rangle_{\text{BS}}}. \tag{S6}$$

Here, E_{HS} and E_{BS} are the ground electronic energies obtained from the high-spin (HS) and broken-symmetry (BS) calculations, and $\langle\hat{\mathbf{S}}^2\rangle_{\text{HS}}$ and $\langle\hat{\mathbf{S}}^2\rangle_{\text{BS}}$ are the expectation values of the magnitude of the total spin.

The obtained isotropic exchange parameters and those from experimental data are tabulated in Table S1. We find B3LYP functionals with 40-50 % of Hartree-Fock exchange well reproduce the the experimental ones.

B. Binding energy vs. photoemission measurements

The binding energies of tris-cyclopentadienyl lutetium (LuCp_3) were estimated and compared with experimental data. The experimental binding energies are taken from the photoelectron spectra (PES) of LuCp_3 in gas phase [S8]. We chose Lu^{3+} ion complex because the $4f$ orbitals are completely filled in the ground state, which can be described by singe Slater determinant. For the calculations, we used the x-ray diffraction structure of CeCp_3 [S9] as the structure of LuCp_3 since their structures are similar to each other and the energy scale of the PES is much larger than the change in energy due to the structure. The binding energies were estimated applying the Koopmans' theorem.

The calculated binding energies are shown in Table S2. In comparison with the experimental data, the B3LYP (20 %) and the HF calculations underestimates and overestimates the $4f$ binding energies, respectively. Better agreement is obtained when the contribution of the HF exchange is about 40 %. Since both the exchange interaction and the binding energies obtained with the hybrid B3LYP functional with about 40 % of the HF exchange are close to the experimental data, we used the functional for the calculations of the transfer Hamiltonian.

III. DERIVATION OF ISING EXCHANGE PARAMETERS FOR LN^{3+} COMPLEXES

In order to project the electronic states from the DFT calculations into the model Hamiltonian (S1), we localized the valence Kohn-Sham orbitals (Pipek-Mezey localization [S10]). The Kohn-Sham orbitals are divided them into the magnetic $4f$ orbitals, the bridging ligand orbitals, and the other ligand orbitals, where the last orbitals are not important for the exchange interaction from each other. The localization on the magnetic core was measured by the Mulliken population on the f type orbitals of the metal centers and the p type orbitals of the bridging ligand. With the use of the localized orbitals as the basis set, we expressed the Kohn-Sham Hamiltonian matrix $\mathbf{h}_{\text{KS}}$ for the magnetic core. Here, the localized f orbitals were transformed into the eigenstates of the atomic orbital angular momentum $\hat{l}_z$ whose quantization axes agree with the *ab initio* main magnetic axes [S1–S3], $|im\sigma\rangle$. On the other hand, the ligand orbitals $|Lr\sigma\rangle$ are chosen so that the bridging ligand part of $\mathbf{h}_{\text{KS}}$ becomes diagonal. Then, the diagonal elements of the Hamiltonian matrix $\mathbf{h}_{\text{KS}}$ are used as the orbital energy levels (the f orbital level is averaged) in Eq. (S2), and the elements in the off-diagonal block matrices between the metals and the bridging ligand as the transfer parameters in Eq. (S3).

The matrix of model Hamiltonian (S1) was calculated using the ground ferromagnetic, the ground antiferromagnetic electron configurations (Fig. 1b in the main text), and the configurations with one-electron transfer between metal sites and between metal and ligand as the basis set. Diagonalizing the Hamiltonian matrix, we obtain the ferromagnetic and antiferromagnetic states. When there is no energy level between these two states, we can project these states into Ising Hamiltonian (Eq. (3) in the main text). The Coulomb repulsion u_f is determined to reproduce the experimental exchange parameter.

The obtained exchange parameters are shown in Table I in the main text. The Coulomb repulsion energies are 1.50, 1.40, 1.20 eV for the Tb, Dy, Ho complexes in the series (a), 1.48 and 1.44 eV for the complexes (b) and (c), respectively. In all cases, there is no level between the ground ferro- and antiferromagnetic levels. At a glance, u_f looks too small (cf. Ref. S11), whereas it is not too small because the effect of the Coulomb repulsion is partly included in the Kohn-Sham orbital energies within the mean-field approximation. Thus, u_f is the difference between the Hubbard U and the mean-field value. The ferromagnetic and the antiferromagnetic ground states are mainly contributed by the electron configurations without electron transfer (the type of Fig. 1b in the main text). Their contributions (probabilities) to the ground state are 98.3 %, 99.4 %, 98.9 % for the Tb, Dy, Ho complexes of the series (a), 99.3 % for the complex (b), 99.7 % for the complex (c), and 97 % for the Er complex. These high probabilities guarantee the validity of the description of the low-lying states by the pseudospin Hamiltonian.

TABLE S1. The exchange parameters extracted from the broken-symmetry DFT calculations and experimental magnetic susceptibility (cm^{-1}). The numbers in the first row indicate the contribution of the HF part in the exchange-correlation functional. The last column are the experimental data (Exp.) extracted using the Lines model or Heisenberg model. The experimental data of the Gd complexes (a), (b), (d) are taken from Refs. S1, S2, and S7, respectively. The effect of the magnetic dipolar interaction is removed only for the complex (a).

	20 %	25 %	30 %	35 %	40 %	45 %	50 %	55 %	60 %	Exp.
(a)	-0.300	-0.268	-0.235	-0.213	-0.194	-0.180	-0.160	-0.150	-0.133	-0.17 [S1]
(b)	-0.319	-0.280	-0.249	-0.231	-0.207	-0.190	-0.173	-0.157	-0.142	-0.210 [S2]
(d)	-1.468	-1.057	-0.785	-0.603	-0.478	-0.385	-0.312	-0.260	-0.222	-0.448 [S7]

TABLE S2. Binding energies (eV). $4f$, Cp (σ), and Cp (π) are the nature of the Kohn-Sham orbitals for the corresponding range of energies. The experimental energies indicate the peaks of the photoelectron spectra [S8].

	B3LYP					
	20 %	25 %	30 %	35 %	40 %	45 %
$4f$	12.47 - 12.59	13.21 - 13.33	13.96 - 14.08	14.71 - 14.82	15.46 - 15.58	16.21 - 16.33
Cp (σ)	9.97 - 11.22	10.26 - 11.59	10.56 - 11.95	10.85 - 12.32	11.14 - 12.68	11.43 - 13.05
Cp (π)	5.24 - 6.71	5.42 - 6.92	5.60 - 7.12	5.78 - 7.33	5.97 - 7.54	6.15 - 7.74
		B3LYP		HF	Exp.	
	50 %	55 %	60 %			
$4f$	16.97 - 17.09	18.04 - 17.68	18.63 - 18.54	23.29 - 23.21	14.30, 15.74	
Cp (σ)	11.73 - 13.42	12.02 - 13.79	12.31 - 18.35	15.92 - 13.77	12.4	
Cp (π)	6.33 - 7.95	6.52 - 8.16	6.70 - 8.37	9.13 - 7.22	7.28, 8.81	

IV. S-S AND S-D EXCHANGE MECHANISMS IN d METAL COMPLEXES

Within the simple two-sites model for the non-collinear doublets in the main text, we calculated the exchange interaction parameter for d metal complexes assuming that the ground state is $|J, \pm J\rangle$. The exchange parameter $\mathcal{J}$ for the d^6, d^7 and d^8 ions are shown in Fig. S1(a). As in the case of the f metal ions, the exchange becomes ferromagnetic as the increase of angle ϕ.

Although the crystal field in transition metal complex is stronger than in lanthanide complex, the crystal field level with unquenched orbital can be obtained with suitable symmetry of the ligands. For example, axial (d^6, d^7) or trigonal (d^8) crystal field splits the d levels into two doublets and one nondegenerate state [S12]. With the splitting, the ground doublets of the d^6 metal originates from the J-multiplet due to the strong Hund's rule coupling (Fig. S1(b)). In the case of the d^7 and d^8 systems, the orbital momentum is unquenched in the presence of the spin-orbit coupling which exceeds the crystal field splitting of the orbital levels (Δ in Fig. S1(b)), which could be observed even in $3d$ metals systems.

[S1] J. Long, F. Habib, P.-H. Lin, I. Korobkov, G. Enright, L. Ungur, W. Wernsdorfer, L. F Chibotaru, and M. Murugesu, "Single-Molecule Magnet Behavior for an Antiferromagnetically Superexchange-Coupled Dinuclear Dysprosium(III) Complex," J. Am. Chem. Soc. **133**, 5319–5328 (2011).

[S2] F. Tuna, C. A. Smith, M. Bodensteiner, L. Ungur, L. F. Chibotaru, E. J. L. McInnes, R. E. P. Winpeny, D. Collison, and R. A. Layfield, "A High Anisotropy Barrier in an Sulfer-Bridged Organodysprosium Single-Molecule Magnet," Angew. Chem. Int. Ed. **51**, 6976–6980 (2012).

[S3] S. K. Langley, D. P. Wielechowski, V. Vieru, N. F. Chilton, B. Moubaraki, L. F. Chibotaru, and K. S. Murray, "Modulation of slow magnetic relaxation by tuning magnetic exchange in $\{Cr_2Dy_2\}$ single molecule magnet," Chem. Sci. **5**, 3246–3256 (2014).

[S4] J. J. Le Roy, L. Ungur, I. Korobkov, L. F Chibotaru, and M. Murugesu, "Coupling Strategies to Enhance Single-Molecule Magnet Properties of Erbium-Cyclooctatetraenyl Complexes," J. Am. Chem. Soc. **136**, 8003–8010 (2014).

[S5] F. Neese, "The ORCA program system," WIREs Comput. Mol. Sci. **2**, 73–78 (2012).

[S6] T. Soda, Y. Kitagawa, T. Onishi, Y. Takano, Y. Shigeta, H. Nagao, Y. Yoshioka, and K. Yamaguchi, "Ab initio computations of effective exchange integrals for H-H, H-He-H and Mn_2O_2 complex: comparison of broken-symmetry approaches," Chem. Phys. Lett. **319**, 223–230 (2000).

[S7] J. J. Le Roy, M. Jeletic, S. I. Gorelsky, I. Korobkov,

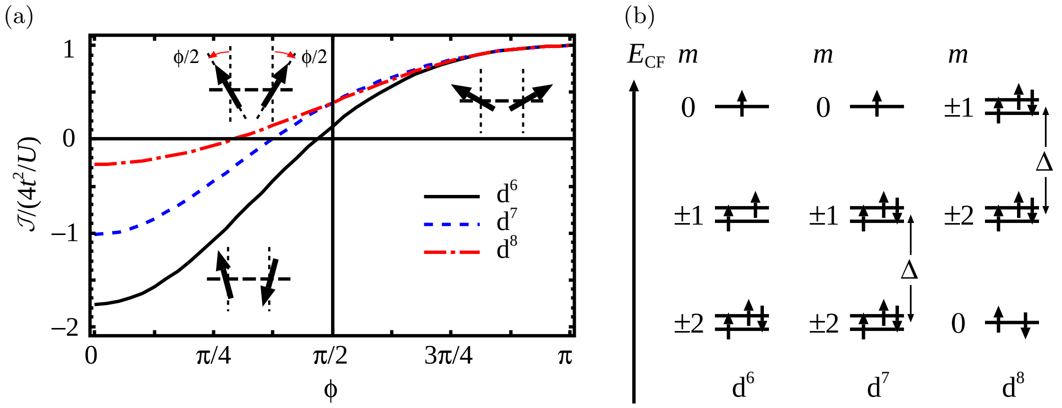

FIG. S1. (a) $\mathcal{J}$ for several d-metal complexes as function of the angle between local main magnetic axes. (b) d^6, d^7 and d^8 electron configurations corresponding to axial doublets with maximal M_J. The non-aufbau population in the last two electron configurations is achieved for relatively small separation Δ between orbital levels with non-zero m (compared to spin-orbit coupling). This order of orbitals is realized, for example, in crystal fields with axial symmetry (d^6, d^7) and/or trigonal symmetry (d^8) [S12].

L. Ungur, L. F Chibotaru, and M. Murugesu, "An Organometallic Building Block Approach To Produce a Multidecker 4f Single-Molecule Magnet," J. Am. Chem. Soc. **135**, 3502–3510 (2013).

[S8] M. Coreno, M. de Simone, R. Coates, M. S. Denning, R. G. Denning, J. C. Green, C. Hunston, N. Kaltsoyannis, and A. Sella, "Variable Photon Energy Photoelectron Spectroscopy and Magnetism of $YbCp_3$ and $LuCp_3$," Organometallics **29**, 4752–4755 (2010).

[S9] U. Baisch, S. Pagano, M. Zeuner, J. Schmedt auf der Günne, O. Oeckler, and W. Schnick, "Synthesis, Structure, and Dynamics of Tris(η^5-cyclopentadienyl)lanthanides and Bis(η^5-cyclopentadienyl)[bis(trimethylsilyl)amido]cerium(III)," Organometallics **25**, 3027–3033 (2006).

[S10] J. Pipek and P. G. Mezey, "A fast intrinsic localization procedure applicable for abinitio and semiempirical linear combination of atomic orbital wave functions," J. Chem. Phys. **90**, 4916–4926 (1989).

[S11] D. van der Marel and G. A. Sawatzky, "Electron-electron interaction and localization in d and f transition metals," Phys. Rev. B **37**, 10674–10684 (1988).

[S12] L. Ungur, M. Thewissen, J.-P. Costes, W. Wernsdorfer, and L. F. Chibotaru, "Interplay of strongly anisotropic metal ions in magnetic blocking of complexes," Inorg. Chem. **52**, 6328–6337 (2013).
